# Evaluation of urethral thickness using ultrasonography in healthy small-breed dogs

**DOI:** 10.3389/fvets.2022.1051898

**Published:** 2022-12-07

**Authors:** Geunha Kim, Yewon Ji, Donghyeok Choo, Sung-Soo Kim, Kichang Lee, Hakyoung Yoon

**Affiliations:** ^1^Department of Veterinary Medical Imaging, College of Veterinary Medicine, Jeonbuk National University, Iksan, South Korea; ^2^VIP Animal Medical Center, Seoul, South Korea

**Keywords:** canine, small-dog, ultrasound, urethra, urethritis, urethral wall, urethral thickness, reference range

## Abstract

**Introduction:**

Urethral thickness measurements can be indicative of the pathological state of a patient; however to the best of our knowledge, no measurement reference range has been established in small-breed dogs. This study aimed to establish reference ranges for total urethral thickness and urethral wall thickness in healthy small-breed dogs; “urethral wall thickness” was assumed to be 1/2 of the “total urethral thickness.”

**Methods:**

Total urethral thickness was measured by ultrasonography in 240 healthy small-breed dogs. In both female and male dogs, the thickness was measured in the mid-sagittal plane. In female dogs, it was measured immediately before the pelvic bone. In male dogs, it was measured caudal to the prostate and cranial to the pelvic bone. The total urethral thickness we measured is the total thickness of the collapsed urethra, which is the sum of the thicknesses of the dorsal and ventral urethral wall.

**Results:**

The mean value of total urethral thickness was 3.15 ± 0.83 mm (urethral wall thickness, 1.58 ± 0.41 mm) in 240 small-breed dogs. The total urethral thickness was significantly greater in male dogs than in female dogs (*p* < 0.001), even when compared among the same breeds (*p* < 0.05). The mean value of the total urethral thickness in females was 2.78 ± 0.60 mm (urethral wall thickness, 1.39 ± 0.30 mm), and 3.53 ± 0.86 mm (urethral wall thickness, 1.76 ± 0.43 mm) in males. There was very weak positive correlation between body weight (BW) and total urethral thickness (R2 = 0.109; β = 0.330; *p* < 0.001). Intraobserver reliability measured by intraclass correlation coefficient (ICC) was 0.986 (*p* < 0.001) and interobserver reliability measured by ICC was 0.966 (*p* < 0.001).

**Discussion:**

This study described the differences in total urethral thickness between breeds, sexes, and sterilization status, and the correlation between BW and total urethral thickness. Furthermore, this is the first study to provide reference ranges of total urethral thickness and urethral wall thickness in small-breed dogs using ultrasonography, and is expected to be useful for urethral evaluation in veterinary diagnostic imaging.

## Introduction

Urethral diseases can cause pollakiuria, stranguria, dysuria, hematuria, pyuria, and urinary tract infections due to immune-mediated inflammation of the urinary tract as well as weight loss, anorexia, and lethargy if the disease condition worsens (1–8). Management with the medical treatment of the disorders secondary to urethral diseases, such as urethral stenosis of urethral obstruction can be difficult. Therefore, surgical treatment might be needed but it often results in post-operative complications such as urethral restricture and can be fatal in severe cases (4, 9, 10). As it can lead to life-threatening conditions and affect the quality of life of patients, an early diagnosis of these disorders and the evaluation of the urethra are essential (11, 12).

In dogs with various urethral diseases, thickening of the urethral wall, narrowing or distending of the lumen, or filling defects can be observed. Most urethral diseases including inflammation, infection, urethral calculi, and tumors, can make the urethral wall thicker and irregular, especially in cases of urethritis and urethral neoplasia (5–7, 10). Furthermore, reduced definition of the urethral wall layers may indicate aggressive conditions, such as severe inflammation or neoplasia (1, 13).

In the past, total urethral thickness was evaluated by palpation on rectal, vaginal or abdominal examination (5–7, 13). However, this method can be subjective and it is not easy to know exactly which location is under the palpation. Evaluation of total urethral thickness using ultrasound was also performed in a previous study but the objective criteria for thickening of the urethral wall have not been clearly established (13). Therefore, quantitative reference ranges for total urethral thickness or urethral wall thickness are needed; urethral wall thickness was assumed to be 1/2 of the total urethral thickness.

Non-invasive or minimally invasive imaging modalities are used for evaluation of the urethra (14–17). Radiography is not available for urethral evaluation because it does not enable visualization of the urethra. On computed tomography (CT) and magnetic resonance imaging (MRI), slice thickness and limited matrix size may decrease the accuracy of total urethral thickness measurements. The slice thickness can cause partial volume averaging artifacts in all cross-sectional imaging modalities because the signal intensity of the pixel is the average of all individual voxel (18, 19). If the urethra is adjacent to the surrounding soft tissue, the margin of the urethral wall may not be clearly visualized. CT and MRI have the disadvantages of being more expensive with relatively longer scanning time. In contrast, ultrasound can clearly visualize the urethral wall even if it is adjacent to the surrounding soft tissue and has the additional benefits of a lower cost, shorter procedure time, not requiring anesthesia, rarely requiring sedation, and avoiding patient or operator exposure to ionizing radiation (14). Therefore, we used ultrasonography to measure total urethral thickness in this study.

The purposes of this study were to: (1) establish a reference range for total urethral thickness and urethral wall thickness in healthy small-breed dogs; (2) analyze the statistical differences in total urethral thickness between breeds, sexes, and sterilization status; and (3) analyze the correlation between body weight (BW) and total urethral thickness.

## Materials and methods

### Animals

This was a multicenter, retrospective and prospective, observational study. The medical records and ultrasonographic images of 364 small-breed dogs were collected from two veterinary clinics. A total of 178 dogs were included in a prospective study from Jeonbuk National University Animal Medical Center between April 2021 and February 2022, and 186 dogs were included in a retrospective study from Ye-eun Animal Medical Center between March 2017 and January 2018. A detailed medical history including breed, sex, age and BW was taken for each dog. For the inclusion criteria, dogs had to be healthy with a BW <10 kg. In addition, dogs without abnormalities in blood and urine analyses, history of urinary tract infection, and clinical signs related to the urinary system, were selected. Dogs not meeting the inclusion criteria and dogs with a pelvic bladder or no apparent urethra, were excluded from this study. Finally, 115 dogs from the prospective study and 125 dogs from the retrospective study were included in the analyses.

This study was approved by the Institutional Animal Care and Use committee of Jeonbuk National University (Approval No. JBNU 2021-0104).

### Measurements

**Figure 1:**
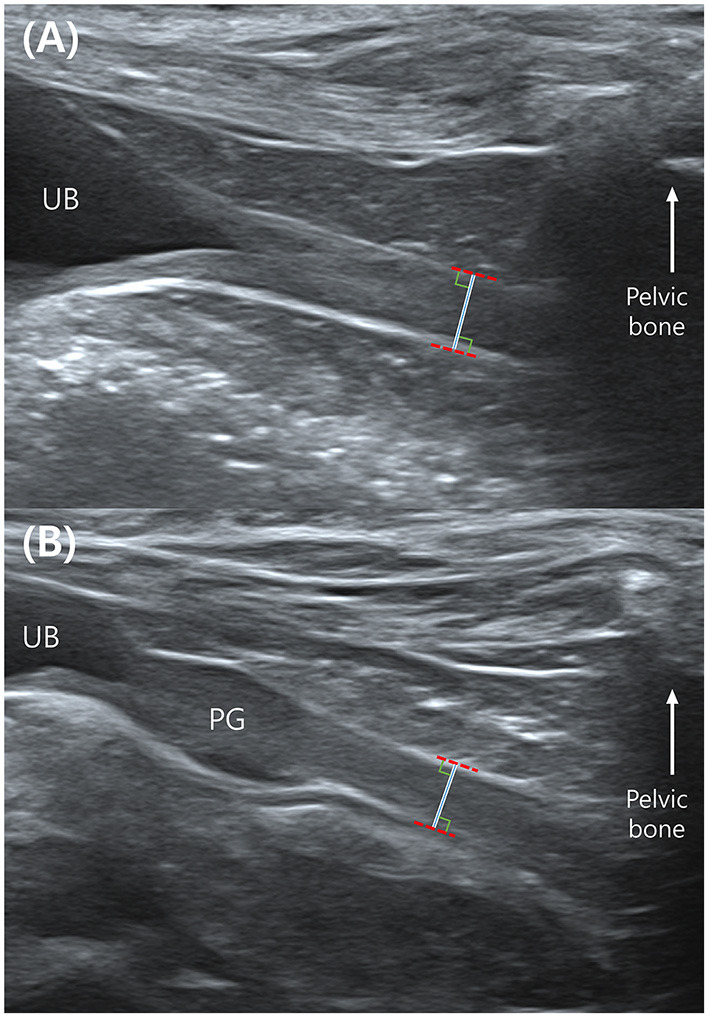
Mid-sagittal plane of the urethra in a female **(A)** and a male dog **(B)** in ultrasonography. The total urethral thickness is measured directly in front of the pelvic bone which makes acoustic shadowing. The measurement is made with the leading edge to the trailing edge. UB, urinary bladder; PG, prostate gland.

Abdominal ultrasound was conducted using a 13-MHz linear array transducer (Aplio 300; Canon Medical System, Europe B.V., Zoetermeer, Netherlands), a 15-MHz linear array transducer (Aplio i800; Canon Medical Systems, Tokyo, Japan), and a 13-MHz linear array transducer (Accuvix XG; Samsung Medison, Seoul, Korea). The ultrasound examination of the urethra was performed by placing the dogs in dorsal recumbency. The urethra was examined in a mid-sagittal plane using a linear probe. To reduce the variation in the measurement location, the total urethral thickness was measured immediately before the point where the urethra was not visible due to acoustic shadowing from the pelvic bone in female dogs. In male dogs, the thickness of membranous urethra was measured caudal to the prostate and cranial to the pelvic bone (Figures 1A,B).

**Figure 2:**
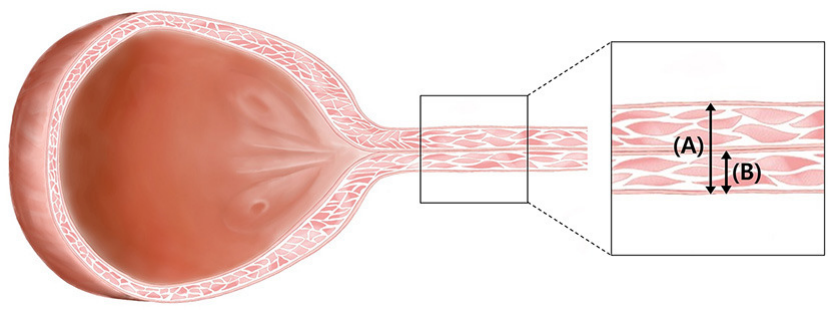
Illustration of urinary bladder and urethra in female dogs. Under normal conditions, the lumen of the urethra is not dilated. It is reasonable to assume that the “total urethral thickness” **(A)** is twice the “urethral wall thickness” **(B)**.

Visual inspection of ultrasound images indicates when the ultrasound beam is perpendicular to the urethra, a normal urethra appears as two thin parallel hyperechoic lines. The urethral lumen and mucosal lining may or may not be visible. When the urethral lumen is distended with urine, the lumen appears anechoic to hypoechoic due to urine and the mucosal lining may be distinguished. However, in most dogs, the urethral lumen is collapsed, and in those cases, the urethral lumen and mucosal lining are not observed in ultrasound images (14). The normal urethral lumen is collapsed without continuous excretion of urine (14); therefore, all dogs included in this study had a collapsed urethral lumen. We measured the distance from the ventral hyperechoic line to the dorsal hyperechoic line (the leading edge to the trailing edge) using electronic calipers (Figures 1A,B). This distance was the thickness of the total urethra that is, assumed to be twice the thickness of the urethral wall. Therefore, the value of the “urethral wall thickness” was assumed to be 1/2 of the measured value, “total urethral thickness” (Figure 2).

For intraobserver reliability analysis, observer A measured the total urethral thickness of 115 dogs (63 females, 52 males) collected in a prospective study two times. For interobserver reliability analysis, the total urethral thickness of these 115 dogs was measured by five clinicians, observer A-E (Residents in the Veterinary Medical Imaging Department of the Teaching Hospital of Jeonbuk National University).

### Statistics

All values are presented as mean and standard deviation. One-way analysis of variance was applied to analyze the differences in total urethral thickness between breeds, sexes, and sterilization status. Linear regression analysis was used to analyze the correlation “between age of sterilization and total urethral thickness,” and “between BW and total urethral thickness.” Intraobserver and interobserver reliability for all measurements was assessed using absolute agreement-type intraclass correlation coefficient (ICC) with 95% confidence intervals (CI). All experimental values were determined to be statistically significant at *p* < 0.05 and highly significant at *p* < 0.001. IBM SPSS Statistics (version 27.0; IBB Corp., Armonk, NY, USA) was used for all the statistical analyses.

## Results

A total of 240 dogs, 120 (50%) females and 120 (50%) males, were included; the mean age of all the dogs was 8.45 (range: 0.3–17.9) years and the mean BW 4.15 (range: 0.9–10) kg. The breeds and sexes of the 240 small-breed dogs were as follows: Maltese (*n* = 103, 52 females, 51 males), Spitz (*n* = 12, 6 females, 6 males), Shih Tzu (*n* = 25, 12 females, 13 males), Yorkshire Terrier (*n* = 20, 10 females, 10 males), Chihuahua (*n* = 13, 6 females, 7 males), Pomeranian (*n* = 26, 13 females, 13 males), and Poodle (*n* = 41, 21 females, 20 males). The mean BW was 3.58 kg in Maltese, 7.84 kg in Spitz, 6.46 kg in Shih Tzu, 2.6 kg in Yorkshire Terrier, 3.32 kg in Chihuahua, 3.29 kg in Pomeranian, and 4.66 kg in Poodle.

Using ultrasound, the urethra just in front of the pelvic bone was identified and observed clearly enough to be measured in 240 healthy small-breed dogs. The mean total urethral thickness was 3.15 ± 0.83 mm. Assuming that the “urethral wall thickness” is half the value of the “total urethral thickness,” the mean urethral wall thickness was 1.58 ± 0.41 mm in all small-breed dogs.

### Significant differences in total urethral thickness between breeds

In seven small-breeds, including Maltese, Spitz, Shih Tzu, Yorkshire Terrier, Chihuahua, Pomeranian, and Poodle, we compared total urethral thickness between the breeds. The total urethral thickness was 3.20 ± 0.92 mm (urethral wall thickness, 1.60 ± 0.46 mm) in Maltese, 3.33 ± 0.86 mm (urethral wall thickness, 1.67 ± 0.43 mm) in Spitz, 3.40 ± 0.87 mm (urethral wall thickness, 1.70 ± 0.44 mm) in Shih Tzu, 2.90 ± 0.55 mm (urethral wall thickness, 1.45 ± 0.27 mm) in Yorkshire Terrier, 3.00 ± 0.81 mm (urethral wall thickness, 1.50 ± 0.40 mm) in Chihuahua, 2.83 ± 0.58 mm (urethral wall thickness, 1.41 ± 0.29 mm) in Pomeranian, and 3.21 ± 0.75 mm (urethral wall thickness, 1.61 ± 0.38 mm) in Poodle. There were no statistically significant differences in total urethral thickness between the breeds (*p* = 0.132). Moreover, considering we found differences in total urethral thickness between the sexes, we compared the measurements among only female dogs, and among only male dogs in all seven breeds. There were no statistically significant differences in total urethral thickness between breeds among females (*p* = 0.177) or males (*p* = 0.497).

### Significant differences in total urethral thickness between sexes

**Figure 3:**
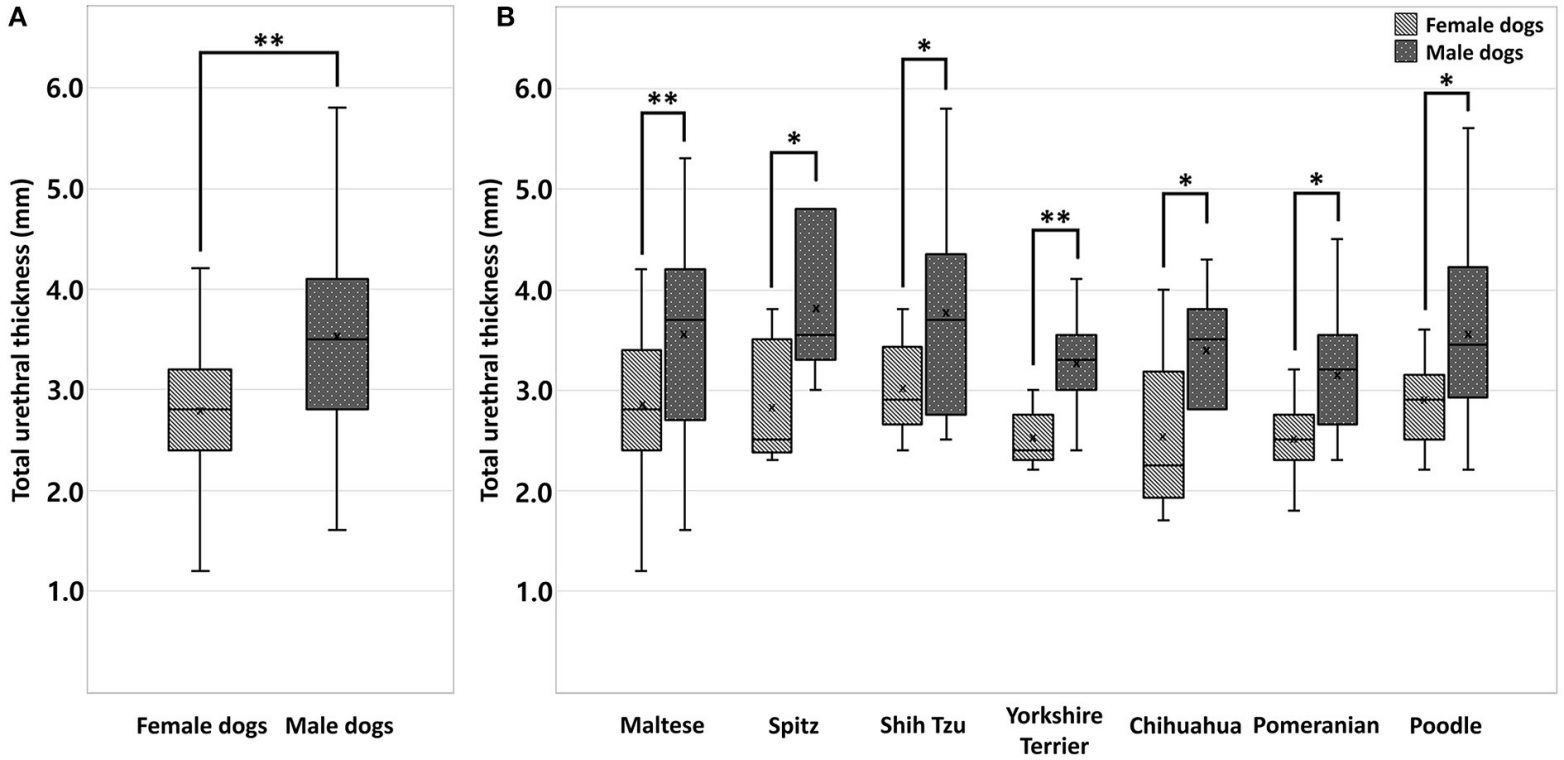
Box-and-whisker plot **(A)** of total urethral thickness ranges in healthy small-breed dogs (*n* = 240); female (*n* = 120) and male dogs (*n* = 120). A statistically significant difference in total urethral thickness was found between the sexes (*p* < 0.001**). Box-and whisker plot **(B)** of total urethral thickness ranges in healthy small-breed dogs (*n* = 240); Maltese (*n* = 103, 52 females, 51 males), Spitz (*n* = 12, 6 females, 6 males), Shih Tzu (*n* = 25, 12 females, 13 males), Yorkshire Terrier (*n* = 20, 10 females, 10 males), Chihuahua (*n* = 13, 6 females, 7 males), Pomeranian (*n* = 26, 13 females, 13 males), and Poodle (*n* = 41, 21 females, 20 males). There were significant differences in total urethral thickness between sexes in the Maltese and Yorkshire Terrier breeds (*p* < 0.001**) and in the Spitz, Shih Tzu, Chihuahua, Pomeranian, and Poodle breeds (*p* < 0.05*).

The 240 dogs were divided into two groups, female dogs (*n* = 120) and male dogs (*n* = 120). A statistically significant difference in total urethral thickness was found between the sexes (*p* < 0.001). The mean total urethral thickness was 2.78 ± 0.60 mm (urethral wall thickness, 1.39 ± 0.30 mm) in female dogs, and 3.53 ± 0.86 mm (urethral wall thickness, 1.76 ± 0.43 mm) in male dogs. The total urethral thickness was significantly greater in male dogs than in female dogs (Figure 3A, Table 1). Even when compared among the same breeds, the thickness of male dogs was significantly greater than that of female dogs in all breeds. There were significant differences in total urethral thickness between sexes in the Maltese and Yorkshire Terrier breeds (*p* < 0.001) and in the Spitz, Shih Tzu, Chihuahua, Pomeranian, and Poodle breeds (*p* < 0.05) (Figure 3B, Table 2).

**Table 1:** Values of total urethral thickness and urethral wall thickness in female (*n* = 120) and male dogs (*n* = 120).

	**Age (years) (range)**	**BW (kg) (range)**	**Mean** ±**SD (mm) (range)**	
			**Total urethral thickness**	**Urethral wall thickness**
Female dogs (*n* = 120)	8.40 (1–17.9)	4.07 (1.52–9.00)	2.78 ± 0.60 (1.2–5.0)	1.39 ± 0.30 (0.6–2.5)
Male dogs (*n* = 120)	8.62 (0.3–16)	4.36 (0.90–10.00)	3.53 ± 0.86 (1.6–6.7)	1.76 ± 0.43 (0.8–3.4)
Total (*n* = 240)	8.45 (0.3–17.9)	4.15 (0.90–10.00)	3.15 ± 0.83 (1.2–6.7)	1.58 ± 0.41 (0.6–3.4)

BW, body weight; SD, standard deviation; Urethral wall thickness, value assumed half the total urethral thickness.

**Table 2:** Values of total urethral thickness and urethral wall thickness in groups divided by breeds and sexes (Maltese, Spitz, Shih Tzu, Yorkshire Terrier, Chihuahua, Pomeranian, and Poodle).

**Breed**	**Sex**	**Mean** ±**SD (mm) (range)**			
		**Total urethral thickness**	**Urethral wall thickness**	**Total urethral thickness**	**Urethral wall thickness**
Maltese (*n* = 103)	F (*n* = 52)	2.84 ± 0.73 (1.2–5.0)	1.42 ± 0.36 (0.6–2.5)	3.20 ± 0.92 (1.2–6.7)	1.60 ± 0.46 (0.6–3.4)
	M (*n* = 51)	3.58 ± 0.95 (1.6–6.7)	1.79 ± 0.48 (0.8–3.4)		
Spitz (*n* = 12)	F (*n* = 6)	2.82 ± 0.62 (2.3–3.8)	1.41 ± 0.31 (1.2–1.9)	3.33 ± 0.86 (2.3–4.8)	1.67 ± 0.43 (1.2–2.4)
	M (*n* = 6)	3.85 ± 0.77 (3.0–4.8)	1.93 ± 0.38 (1.5–2.4)		
Shih Tzu (*n* = 25)	F (*n* = 12)	3.01 ± 0.46 (2.4–3.8)	1.50 ± 0.23 (1.2–1.9)	3.40 ± 0.87 (2.4–5.8)	1.70 ± 0.44 (1.2–2.9)
	M (*n* = 13)	3.76 ± 1.02 (2.5–5.8)	1.89 ± 0.51 (1.3–2.9)		
Yorkshire Terrier (*n* = 20)	F (*n* = 10)	2.50 ± 0.27 (2.2–3.0)	1.25 ± 0.14 (1.1–1.5)	2.90 ± 0.55 (2.2–4.1)	1.45 ± 0.27 (1.1–2.1)
	M (*n* = 10)	3.29 ± 0.46 (2.4–4.1)	1.65 ± 0.23 (1.2–2.1)		
Chihuahua (*n* = 13)	F (*n* = 6)	2.52 ± 0.83 (1.7–4.0)	1.26 ± 0.41 (0.9–2.0)	3.00 ± 0.81 (1.7–4.3)	1.50 ± 0.40 (0.9–2.2)
	M (*n* = 7)	3.41 ± 0.55 (2.8–4.3)	1.71 ± 0.28 (1.4–2.2)		
Pomeranian (*n* = 26)	F (*n* = 13)	2.51 ± 0.34 (1.8–3.2)	1.25 ± 0.17 (0.9–1.6)	2.83 ± 0.58 (1.8–4.5)	1.41 ± 0.29 (0.9–2.3)
	M (*n* = 13)	3.15 ± 0.59 (2.3–4.5)	1.57 ± 0.30 (1.2–2.3)		
Poodle (*n* = 41)	F (*n* = 21)	2.89 ± 0.41 (2.2–3.6)	1.45 ± 0.20 (1.1–1.8)	3.21 ± 0.75 (2.2–5.6)	1.61 ± 0.38 (1.1–2.8)
	M (*n* = 20)	3.55 ± 0.89 (2.2–5.6)	1.77 ± 0.44 (1.1–2.8)		

In each breed, the mean value of male dogs was significantly greater than that of female dogs.

F, female; M, male; SD, standard deviation; Urethral wall thickness, value assumed half the total urethral thickness.

#### Significant differences in total urethral thickness between sterilization status

The comparison between the sexes was conducted without considering whether sterilization was performed, and we investigated the effects of sterilization status on total urethral thickness, considering that sterilization may have affected the results. To perform the analysis, we divided the dogs into female (77 spayed, 43 intact) and male (109 castrated, 11 intact) groups. There was no statistically significant differences in total urethral thickness between the spayed female dogs and the intact female dogs (*p* = 0.76) or between the castrated male dogs and the intact male dogs (*p* = 0.44).

#### Correlation between sterilization ages and total urethral thickness

To determine the correlation between total urethral thickness and age of sterilization, 71 dogs with known sterilization ages were analyzed. There was no correlation between total urethral thickness and sterilization age (linear model, *R*^2^ = 0.000; β = 0.002; *p* = 0.988).

### Correlations between BW and total urethral thickness

Furthermore, we investigated the effects of BW on total urethral thickness in all dogs. There was very weak positive correlation between BW and total urethral thickness (*R*^2^ = 0.109; β = 0.330; *p* < 0.001) in all dogs, female (*R*^2^ = 0.092; β = 0.303; *p* < 0.05) or male (*R*^2^ = 0.104; β = 0.323; *p* < 0.001).

### Intraobserver and interobserver reliability

The mean total urethral thickness of 115 dogs (63 females, 52 males) collected in a prospective study was 3.52 ± 0.86 mm in the first measurement of observer A. The measurement was repeated once more by observer A and the mean total urethral thickness was 3.51 ± 0.81 mm. The median ICC showed almost perfect reliability for the two measurements. Intraobserver reliability measured by ICC was 0.986 [95% CI: 0.979–0.990, *p* < 0.001] (Table 3).

**Table 3:** Intraobserver (observer A) reliability for the total urethral thickness measurements of 115 dogs using ICC and their 95% CI.

**Repetition**	**Mean ±SD (mm)**	**ICC**	**95% CI**	***P*-value**
1	3.52 ± 0.86	0.986	0.979–0.990	<0.001
2	3.51 ± 0.81			

SD, standard deviation; ICC, intraclass correlation coefficient (absolute agreement); CI, confidence interval.

The total urethral thickness of these 115 dogs was measured by five clinicians, observer A–E (observer A, 3.52 ± 0.86 mm; observer B, 3.50 ± 0.87 mm; observer C, 3.39 ± 0.88 mm; observer D, 3.52 ± 0.91 mm; and observer E, 3.49 ± 0.86 mm). The median ICC showed almost perfect reliability for the five measurements. Interobserver reliability measured by ICC was 0.966 [95% CI: 0.954–0.975, *p* < 0.001] (Table 4).

**Table 4:** Interobserver reliability for the total urethral thickness measurements of 115 dogs using ICC and their 95% CI.

**Interobserver**	**Mean ±SD (mm)**	**ICC**	**95% CI**	***P*-value**
Observer A	3.52 ± 0.86	0.966	0.954–0.975	<0.001
Observer B	3.50 ± 0.87			
Observer C	3.39 ± 0.88			
Observer D	3.52 ± 0.91			
Observer E	3.49 ± 0.86			

SD, standard deviation; ICC, intraclass correlation coefficient (absolute agreement); CI, confidence interval.

## Discussion

This study determined reference ranges for total urethral thickness and urethral wall thickness using ultrasonography in healthy small-breed dogs based on a larger number of samples than previous studies.

To date, there have not been veterinary studies on reference ranges for total urethral thickness or urethral wall thickness in small-breed dogs. In a recent study, although their purpose was not to make a reference range of the urethral wall thickness, the thickness of the urethral wall of 10 male Beagle dogs was measured by conventional ultrasound without contrast injection. In the previous study, the mean value of the urethral wall thickness in membranous urethra was 1.4 ± 0.3 mm (14). This value is smaller than in our study, but there are differences in measurement location and method between the two studies. In the previous study, the membranous urethra was measured just proximal to the greater curvature. In addition, only hypoechoic part was measured in the previous study, but we measured the urethral wall thickness including hyperechoic lines. Therefore, different reference ranges of urethral wall thickness can be applied depending on the measurement location and method.

In our study, the thickness of total urethra was measured using ultrasound. Ultrasound allows direct visualization of margins. On ultrasonography, the urethra of the female dog can be scanned, except for the caudal portion of the intrapelvic area. In most male dogs, the prostatic urethra and penile urethra can be visualized, but only a portion of the membranous urethra can be visualized due to acoustic shadowing from the pelvic bone.

When selecting the measurement location in female dogs, the location with the least variation was considered. The location where the urethral thickness is measured should be where the urethral lumen is not distended to avoid overestimation of the thickness since it is measured from the ventral margin to the dorsal margin of the urethra. As a result of retrograde urethrography in one study, the degree of bladder distention had an effect on the lumen distention of the proximal urethra, but had no significant effect on the distal urethra in both female and male dogs (17). Another study also revealed through CT urethrography that the closer to the proximal side, the more affected the urethral lumen diameter with the distention of the bladder in female dogs (20). This result may have been influenced by the smaller muscle mass of the proximal urethra compared to the distal urethra (20, 21). Based on these studies, the most distal urethra may have the least effects depending on the degree of distention of the bladder. Since the distal urethra has a poor window for ultrasonography due to acoustic shadowing from the pelvic bone, the location we opted for was immediately before the pelvic bone in the distal urethra.

In male dogs, we measured the thickness of the membranous urethra before the pelvic bone. In a study which performed retrograde urethrography in dogs, the degree of bladder distention had significant effect on the lumen distention of the prostatic urethra, but had no significant effect on the membranous and penile urethra (17). In another study using CT urethrography, male dog urethra was divided into five sites (cranial prostatic urethra, middle prostatic urethra, caudal prostatic urethra, membranous urethra, and penile urethra), and it was revealed that diameters of the caudal prostatic urethra and the membranous urethra showed no significant difference between the empty and distended bladder (22). In addition, because the prostatic urethra is surrounded by fibroelastic tissue and no sphincteric smooth muscle, it might be susceptible to hydrostatic pressure of the fluid in the urethral lumen (22, 23). Furthermore, the prostatic urethra appears as a hypoechoic area between two symmetrical lobes on ultrasound, but the boundary may be ambiguous because the hyperechoic wall may or may not be visible (24, 25). We considered the prostatic urethra to be an inappropriate location for measuring total urethral thickness due to its histological structure and ultrasonographic features. Therefore, the location we opted for was the membranous urethra in male dogs.

Additionally, total urethral thickness was not measured in a transverse plane because it is difficult to know exactly where the current view is located in the urethra in a transverse plane. Furthermore, because the urethra does not run parallel to the abdominal wall, it is difficult to make it perpendicular to the transverse plane. Therefore, it is recommended to measure total urethral thickness in a mid-sagittal plane than a transverse plane.

The differences in total urethral thickness between breeds were compared. The Yorkshire Terrier, Chihuahua, and Pomeranian breeds tended to have smaller total urethral thickness compared to other breeds but there was no statistically significant difference. Since these breeds have relatively lower body weights, this result was understood to be due to a positive correlation between BW and total urethral thickness.

Total urethral thickness was significantly thicker in male dogs compared to female dogs. This is considered to be due to differences in anatomical structures. In female dogs, the proximal half to two-thirds of the urethra is surrounded by circular smooth muscle, and in the distal one-third to half of the urethra, striated muscle (urethralis muscle) replaces smooth muscle (21, 26). In male dogs, the post-prostatic urethra is encircled by a thick coat of striated muscle (urethralis muscle), which starts from the caudal prostatic region (23, 26).

Therefore, considering that there was no difference in total urethral thickness between small-breeds included in this study and that there was a difference in total urethral thickness between sexes, reference ranges of total urethral thickness in female and male dogs were established. In addition, considering that the urethral wall thickness does not change significantly depending on the degree of urethral distention, the reference ranges can be applied to various states, with or without dilation of the urethral lumen, unlike the urinary bladder wall which varies depending on the degree of bladder distention (14, 27).

Sterilization status and sterilization ages had no significant effect on total urethral thickness in both female and male dogs. The correlation between sterilization and total urethral thickness has not been clearly known in previous studies in dogs. In this study, there is a possibility that the results for male dogs may not have been accurate due to the small number of included intact male dogs; the number of castrated male dogs was 109, whereas the number of intact male dogs was 11.

There was very weak correlation between BW and total urethral thickness. The fact that the dogs used in the analyses have similar BW may have led to the results. However, considering that there is a very weak but positive correlation, BW may significantly affect total urethral thickness in dogs with various BW. It is judged that further study using dogs with various BW is necessary.

In intraobserver and interobserver reliability analyses, we used ICC with 95% CI. ICC is a common method for estimating reliability in different settings. We used ICC based on an absolute agreement to compare absolute values of total urethral thickness. We used interpretation of intraclass correlation coefficient according to Fleiss (28) and Viera and Garrett (29). According to the interpretation, we considered that ICC value (a) < 0 (less than chance agreement), (b) 0.01–0.20 slight agreement, (c) 0.21–0.40 fair agreement, (d) 0.41–0.60 moderate agreement, (e) 0.61–0.80 substantial agreement, and (f) 0.81–0.99 almost perfect agreement. Intra- and interobserver reliability analyses showed almost perfect agreement, with an ICC of 0.986 [95% CI: 0.979–0.990, *p* < 0.001] in intraobserver reliability analysis and an ICC of 0.966 [95% CI: 0.954–0.975, *p* < 0.001] in interobserver reliability analysis. Based on the results, it was considered that there was no significant error in the measurement method of the total urethral thickness we set.

Our results should be considered within the context of the study limitations. First, urethral biopsy was not performed in the present study because it is invasive and also have risk of complications (30). To compensate for this, follow-up tests were conducted for up to 3 months to confirm that no urethra or bladder-related diseases occurred after this study. Second, the points at which total urethral thickness being measured may have varied depending on the degree of bladder distension. In dogs, the urinary bladder lies cranial to or within the pelvic canal when it is empty and extends cranially along the ventral abdominal wall as it distends (31). However, even if the location was changed, the thickness difference was not significant. Third, the total sample of small-breed dogs was large scale, but there were also breeds with small sample size. Evaluation with a larger sample size may be required for each breed. Finally, the urethra in the pelvic canal region could not be measured. It was impossible to measure the urethra in the pelvic cavity using ultrasonography due to the acoustic shadowing from the pelvic bone. CT and MRI can compensate for these problems and detect lesions in regions not observed on ultrasound. In order to study the total urethral thickness in various locations, it is considered that further studies using CT or MRI are necessary.

In conclusion, to the best of our knowledge, this was the first study to establish reference ranges of total urethral thickness and urethral wall thickness using ultrasonography in healthy small-breed dogs. Since there was no significant difference in total urethral thickness between the breeds, the same reference range can be applied in Maltese, Spitz, Shih Tzu, Yorkshire Terrier, Chihuahua, Pomeranian, and Poodle. In addition, there was a statistically significant difference in total urethral thickness between female and male dogs; therefore, a reference range for female and male dogs was established separately. The mean total urethral thickness was 2.78 ± 0.60 mm in female dogs, 3.53 ± 0.86 mm in male dogs, and 3.15 ± 0.83 mm in all small-breed dogs. Considering the value of the “urethral wall thickness” was assumed to be 1/2 of the “total urethral thickness,” the mean urethral wall thickness was 1.39 ± 0.30 mm in female dogs, 1.76 ± 0.43 mm in male dogs, and 1.58 ± 0.41 mm in all small-breed dogs. These reference ranges are expected to help evaluate the urethra in small-breed dogs.

## Data availability statement

The original contributions presented in the study are included in the article/supplementary material, further inquiries can be directed to the corresponding author.

## Ethics statement

The animal study was reviewed and approved by Institutional Animal Care and Use Committee of Jeonbuk National University (Approval No. JBNU 2021-0104). Written informed consent was obtained from the owners for the participation of their animals in this study.

## Author contributions

Conception and design, acquisition of data, and drafting the article: GK and HY. Analysis and interpretation of data, revising article for intellectual content, and final approval of the completed article: GK, YJ, DC, S-SK, KL, and HY. Agreement to be accountable for all aspects of the work ensuring that questions related to the accuracy or integrity of any part of the work are appropriately investigated and resolved: GK, KL, and HY. All authors contributed to the article and approved the submitted version.
